# Obesity and the possibility of conceiving a child during assisted reproduction treatment: An Argentinian experience

**DOI:** 10.5935/1518-0557.20190064

**Published:** 2020

**Authors:** Juan Carlos Sánchez Páez, Vanina Góméz Arreseygor, Pía Zgrablich

**Affiliations:** 1 Laboratorio de embriología clínica, Centro de Reproducción Asistida GESTAR; 2 Unidad de Medicina reproductiva, Centro de Reproducción Asistida GESTAR

**Keywords:** assisted reproduction technologies, case-control study, BMI, obesity, pregnancy outcome

## Abstract

**Objective:**

The goal of this study was to assess the association between BMI and the possibility of conceiving a child through an assisted reproduction treatment.

**Methods:**

A study of cases and controls matched by age, with 394 patients that underwent treatment at GESTAR (assisted reproduction center), between 2013-2017. The association between BMI and the possibility of conceiving a child, analyzed through logistic regression.

**Results:**

Among the cases (successful treatments) 14% were obese, while in the control group (patients that did not get pregnant) the obesity rate was 21%. There was a significant difference (*p*<0,01) in the BMI, the number of recovered oocytes, normally fertilized oocytes and the number of transferred embryos. The Odds Ratio (OR) in SPSS was 0.26 ± (0.14, 0.50) - 95% CI, indicating that conceiving a child by assisted reproduction is 74 times lower in patients that are obese when compared to non-obese patients (*p*<0,001). And the Odds Ratio (OR) calculated by logistic regression in Stata 11 was 0.80 ± (0.76, 0.86), 95% CI, which indicates a 20% decrease in the possibility of conceiving for each point on the BMI scale.

**Conclusion:**

Obesity is associated with a lower conception likelihood through assisted reproduction technologies.

## INTRODUCTION

Overweight women have higher incidences of anovulation and menstrual dysfunction, possibly caused by hormonal alterations, such as elevated secretion of gonadotropin releasing hormone, luteinizing hormone and androgens. There is also high levels of sexual hormones fixing globulin and insulin resistance (Hamilton-Fairley *et al.,* 1992; Wang *et al.,* 2000; Barrios-De-Tomasi *et al.,* 2013). In reproductive medicine, obesity (Body Mass Index>30Kg/m^2^) has been frequently associated with menstrual disorders, subfertility, involuntary loss and obstetric complications (Bellver *et al.,* 2006; Ben-Haroush *et al.,* 2018).

In Latin America the prevalence of obesity in women that undergo assisted reproduction treatment (ART) is high, 42.4%. These patients are less susceptible to respond to clomiphene citrate, they require higher doses of gonadotropins during ovarian stimulation; they have higher cancellation rates and a lower number of retrieved oocytes, affecting the probabilities of clinical pregnancy or live births (Hamilton-Fairley *et al*., 1992; MacKenna *et al*., 2017). A study from Bellver *et al*. (2003) proved that the rate of spontaneous abortion is higher in obese women receptors of embryos formed by donated ovules. In Argentina, the prevalence of obesity is around 14% according to Elgart *et al*. (2010). But, it is unknown how much the possibility of conceiving a child decreases as the patient's BMI increases.

The goal of this study was to assess the association between BMI and the likelihood of conceiving a child through assisted reproduction. Hypothesis: If obesity is associated with subfertility and failure in reproductive treatments, obese patients that receive treatment will have a decreased possibility of conceiving a child compared to patients with normal BMI.

## MATERIAL AND METHODS

This was a study of cases and controls matched (1:1) by age, with patients that underwent ART (IVF and ICSI); between 2013 and 2017 at the assisted reproduction center GESTAR, located in Buenos Aires, Argentina. A total of 394 patients with the same stimulation protocol, were studied, of which 197 were cases, meaning patients that met the inclusion criteria having at least one live birth from ART; and 197 formed the control group: randomly selected patients with matching ages, without knowing the BMI that received ART without getting pregnant.

Inclusion criteria: Patients that underwent high complexity treatments (IVF or ICSI), between 2013 and 2017 in GESTAR, with their own oocytes, without previous treatments, ages ranging between 24 and 44, that transferred one or two fresh embryos, without taking into account if they had frozen or not the excess embryos.

Exclusion criteria: Patients with incomplete clinical history (age, weight, height, or birth confirmation), patients that presented biochemical pregnancy or spontaneous pregnancy loss.

The data registered for the study included the patients' date of birth and age, number of oocytes obtained, the number of normally fertilized oocytes and the number of transferred embryos; the weight (kg) and height (m). The BMI of every patient was calculated dividing their weight in kilograms by their height in meters elevated to the square (Kg/m^2^). The patients that participated in the study were characterized, according to the four BMI categories proposed by the WHO (WHO, 2004): BMI ≤ 18.4kg/m^2^ (low weight), 18.5-24.9 kg/m^2^ (normal weight), BMI 25-29.9 kg/m^2^ (overweight) and BMI ≥30kg/m^2^ (obese). At the same time these categories turned into two groups, obese patients and non-obese patients.

To prove the difference between the mean value of the BMI groups, number of oocytes retrieved, number of mature oocytes (M2) and number of normally fertilized oocytes, a hypothesis test was made, based on the Student's t-test.

As a measurement of association between the BMI and the possibility of conceiving a child, we calculated the Odds ratio (OR) using logistic regression. The statistical analysis was made using the statistical package for social Sciences, version 10.0 (SPSS Inc., 2000) and the Stata Statistical Software: Release 11 (StataCorp. 2009). The categorical data was expressed as a number and as a percentage, and the numerical data as a mean value and standard deviation.

## RESULTS

Of the 394 patients studied, only 14% were obese (IMC >30Kg/m^2^), specifically 21% of the control group (patients that didn't achieve pregnancy) and 7% of the cases (patients that had a child product of ART with own ovules and fresh transfer). The average age was the same in both groups (35 years ± SD=4) since the controls were matched by age. The most frequently diagnosed infertility factor was a diminishment in the ovarian reserve

**Figure 1:**
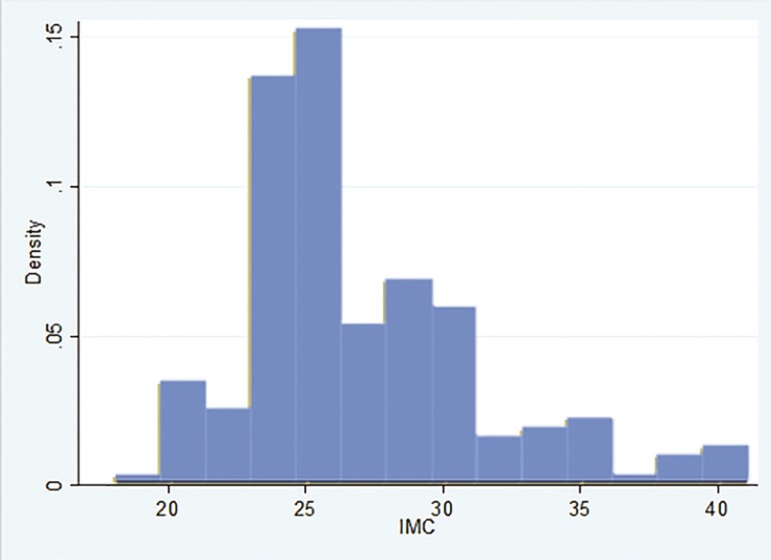
Body Mass Index distribution of Control individuals

**Figure 2:**
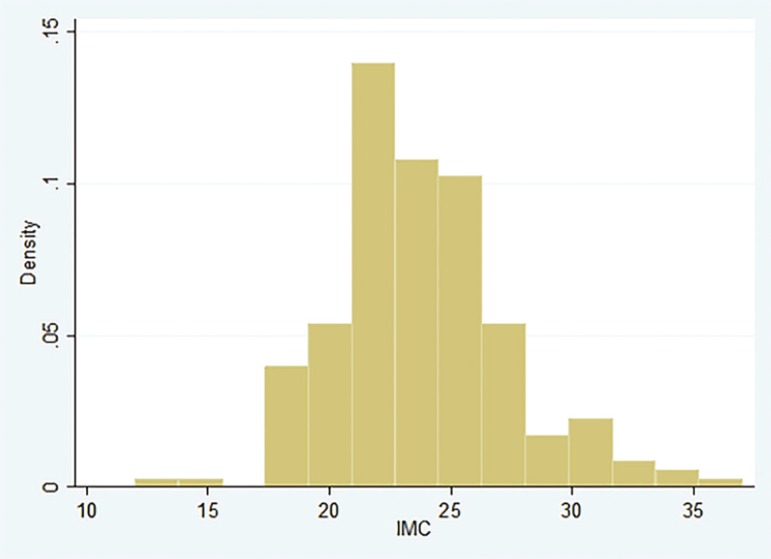
Body Mass Index distribution among the cases

The greater proportion of the patients that achieved having a child through ART (Cases), had a BMI between 20 and 25 kg/m^2^. While among controls, the highest proportion of patients had a BMI between 25 and 30 kg/m^2^, as depicted in Figures 1 and 2.

A significant difference was seen in the BMI, the number of oocytes retrieved, the number of mature oocytes (M2) and the number of normally fertilized oocytes between the groups (*p*<0.01). The characteristics of both groups are shown in Table 1.

**Table 1:** Characteristics by groups of the cases and controls

	CASES	CONTROLS
Infertility factor linked	Low ovarian reserve	Low ovarian reserve
BMI [Mean ± SD)	23.76±4.2	26.93±3.8*
Oocytes retrieved [Mean ± SD)	8.2±6.3	6.6± 4.6*
Oocytes M2[Mean ± SD)	6.2±3.5	4.7±3.3*
Normally fertilized [Mean ± SD)	4.9±2.7	3.6±2.6*
Embryos transferred [Mean ± SD)	2.0±0.5	2.0±0.6

Values are expressed as % (n) or mean ± standard error.

* *p*<0.01 compared with Cases.

The BMI between patients who managed to conceive and those who did not get pregnant differs 3.16 BMI points. Patients who did not get pregnant were more obese than those who got pregnant. They had less mature oocytes recovered, and had lower number of normally fertilized oocytes.

The Odds Ratio (OR) in SPSS was 0.26± (0.14, 0.50) CI 95%, which indicates that conceiving a child by assisted reproduction is 74 times lower in patients that are obese, when compared to non-obese patients (*p*<0,001).

The Odds Ratio (OR) calculated by logistic regression in Stata 11 was 0.80 ± (0.76, 0.86) 95% CI, which indicates a 20% decrease in the possibility of conceiving for each point on the BMI scale (*p*<0.01).

## DISCUSSION

Obesity, including moderate obesity, negatively impacts women's response to ART (Hamilton-Fairley *et al*., 1992; Bellver *et al*., 2003; Dağ & Dilbaz, 2015). In the same way, low weight would have equal effects according to Wittemer *et al.* (2000). However, the data about underweight women is not as consistent as the data from obese women (Bellver *et al.,* 2006).

In our studied population, obesity was 14%, similar to that reported by Elgart *et al*. (2010). High BMI is associated with a lower number of retrieved oocytes, which affects the probabilities of clinical pregnancy, similar to the findings by Moragianni *et al*. (2012), and contrary to what the study by Schliep *et al*. (2015) showed.

A BMI >30 kg/m^2^ is associated with a lower possibility of having a live birth child. Other authors like Bellver *et al.* (2006), Zain & Norman (2008), Brewer & Balen (2010), MacKenna *et al.* (2017) and Ben-Haroush *et al*. (2018) agree that a BMI greater than 30Kg/m^2^ is associated with a significant reduction in the results from assisted reproduction treatments.

Amongst the mechanisms by which the body mass affects reproduction is the decrease in ovarian response to the gonadotropins (Dokras *et al.,* 2006), alterations in the menstrual cycle and anovulation (Barrios-De-Tomasi *et al.,* 2013); however, these problems can be easily overcome with an adequate ovarian stimulation during ART (Bellver *et al.,* 2006). Metwally *et al.* (2007) reported that obesity is associated with lower embryo quality, while other authors like Bellver *et al.* (2006) pointed out the lack of consensus about the impact of obesity in the quality of oocytes and embryos. However, we did not investigate whether the body mass index affects embryo quality; thus we cannot add information in that aspect.

In the absence of differences between the number of transferred embryos in both groups, we suspect that other mechanisms, besides embryo quality can impact treatment success, such as the receptivity of the uterus due to endometrium alterations, as proposed by Wang *et al.* (2000); perhaps the hyper-estrogenic state found in obese women has a negative repercussion in their endometrium receptivity (Barrios-De-Tomasi *et al.*, 2013). Another factor that could be interfering is abdominal fat hampering ultrasound scan during the embryo transfer.

## CONCLUSIONS

In conclusion, whatever the underlying cause is, obesity appears to be a risk factor associated with a decrease in the likelihood of success in having a child born alive through assisted reproduction treatment. There is about 20% decrease in the possibility of conceiving for each point of increase in the BMI scale.

Further studies are required to explain in a prospective way the confounding variables that are associated with an unfavorable response of obese patients to assisted reproduction treatments, for example, the effect of obesity in the quality of the transferred embryos. Obese patients should be educated on the importance of weight loss prior to treatment in order to increase the likelihood of achieving a successful treatment.
